# Aberrant effect of genistein on placenta development expressed through alteration in transforming growth factor-β1 and alkaline phosphatase across the maternal serum, the placenta and the amniotic fluid

**DOI:** 10.22038/ijbms.2020.42493.10022

**Published:** 2020-10

**Authors:** Funmileyi Olubajo Awobajo, Titilola Aderonke Samuel, Ayodele Olufemi Morakinyo, Oluwakemi Tinuolaoluwa Oyelowo, Perpetual Uzoamaka Onyekwele, Ejike Frank Medobi, Mariam Wuraola Abdul, Bilikisu Bukola Aminu, Elo Onome Oruade

**Affiliations:** 1 Department of Physiology, Faculty of Basic Medical Sciences, University of Lagos, Nigeria; 2 Department of Biochemistry, Faculty of Basic Medical Sciences, University of Lagos, Nigeria

**Keywords:** Alkaline phosphatase, Fetal development, Genistein, Placenta, TGf-β1, Trophoblast

## Abstract

**Objective(s):**

The mechanism via which genistein, the major isoflavone content of soya, adversely influenced placenta and fetal development was evaluated in pregnant laboratory rats.

**Materials and Methods:**

There were control, 2 mg/kg and 4 mg/kg genistein groups of rats with five sub-groups based on gestation termination day. At the end of the experiment, animals were sacrificed by CO_2_ and cervical dislocation, while plasma and serum were processed and stored. The abdomen was opened and the amniotic fluid was siphoned from the uterine sacs, processed and stored. The embryonic implants were excised, the placenta was separated from the fetus and weighed separately. Placenta homogenate was prepared from the harvested placenta, while the rest were processed for histological studies. Transforming growth factor (TGf-β1) and alkaline phosphatase (ALP) were assayed for in all samples. A significant decrease in the placenta and fetal weights, and a significant decrease in serum and placenta homogenate ALP levels were recorded in genistein groups.

**Results:**

There was a reduction in the Trophoblast giant cells population (TGCs). TGCs zone depth, perimeter, and an increase in the placenta and amniotic fluid’s TGf-β1 in all genistein groups at GD-13 towards term, and GD-18 and GD-20, respectively. Maternal plasma TGf-β1 was increased in 2 mg group early in pregnancy while its level significantly decreased in both 2 mg and 4 mg genistein groups at mid-gestation towards GD-19.

**Conclusion:**

Genistein aberrant effect on fetal development was via its adverse effect on TGCs proliferation and TGf-β1 activities in the placenta tissue.

## Introduction

The placenta, which is the first organ to be formed at about gestational day four in rodents during embryogenesis is saddled with the responsibility of secreting hormones responsible for sustenance of pregnancy, immunological functions, vascularization, and circulatory functions for easy exchange of nutrients, gaseous materials, and the elimination of metabolic waste products generated (1, 2). The trophoblast cell lineage generates the epithelial part of the placenta and contains several specialized subtypes that play important roles either in altering maternal physiology and blood flow to promote fetal growth or in nutrient uptake (2, 3). Of importance among the trophoblast cell lineage are the Trophoblast giant cells (TGCs), which are the first terminally differentiated cell types to form during embryogenesis in rodents with the capability of supporting embryo implantation and promoting maternal adaptations to pregnancy (1, 3).

The placenta activities are self-regulated in addition to regulation from the maternal endocrine system (4). Among such self-regulation is the secretion of placenta growth factor (PGF) by the trophoblast cells (5) and Transforming growth factor (TGf) secreted from the decidua cells. Although there are conflicting reports on the involvement of Transforming growth factor-β1 (TGf-β1) a variance of the TGf during placentation, there are however strong indications, implicating TGf-β1 with central roles in cell cycle signaling arrest and apoptosis in normal cellular differentiation in such complications of placenta functions such as preeclampsia and fetal growth restriction (6, 7). TGf-β1 is a member of a large multifunctional superfamily of cytokines known to be involved with the regulation of trophoblast cell invasion during implantation of blastocytes and angiogenesis in the uterus (8, 9). Alkaline phosphatase (ALP) on the other hand is an enzyme found in several tissues throughout the body including placenta (10). Elevated blood ALP level is commonly caused by liver disease or bone disorders (11). However, elevated placenta and the amniotic fluid level of ALP have been identified in preterm delivery, placental insufficiency, and low birth weight and thus can serve as a good biomarker of placenta function (12, 13).

Genistein is a naturally occurring estrogen agonist belonging to a class of isoflavones with a higher concentration in soybeans (14). Genistein has been shown to inhibit protein tyrosine kinase and topoisomerase-II activity (5, 16), leading to numerous effects on diverse cell functions. Genistein has been reported to inhibit tumor cell proliferation, trigger cell cycle arrest, and apoptosis (17-20). Pregnant rodents exposed to genistein experienced interrupted implantation process, decreased placenta growth, disruption in the expected pregnancy hormonal milieu with a resultant reduced anogenital distance, and decreased litter birth weight with inflammatory and oxidative stress imbalances (21-24). The mechanisms by which genistein produced these aberrant effects on fetal development are still poorly understood. Therefore, this study aimed to further investigate the influence of genistein on placenta development by quantifying the TGCs population, length, and the thickness of the layer occupied and the levels of ALP and TGf-β1in pregnant rats exposed to genistein.

## Materials and Methods

***Animals and genistein administration***

Observations were made on forty adult female rats, weighing 190 to 200 g. The animals were housed in standard cages and provided clean water and rat feed *ad libitum. *The estrous cycles of all animals were carefully monitored using the vaginal smear technique for three weeks. Animals with regular estrous cycles lasting for an average of four days were mated at a ratio of two females to one matured male, weight-matched after careful observation using the vaginal smear technique. The vaginal smears of the mated rats were checked the following morning under the microscope and the presence of sperm cells confirmed mating while persistent diestrous confirmed pregnancy. The pregnant rats were randomly dived into control, 2 mg, and 4 mg genistein-treated groups. The pregnant rats were randomly divided into groups at a population of 30 rats per group and 6 rats per sub-group. Genistein (purity 98.2%) was purchased from Chengdu Biopurify Phytochemicals ltd. (China). Genistein was stored at - 4 °C and daily preparation was made by weighing and sonication in a predetermined volume of distilled water. Preparations were made daily to avoid decay of genistein. Genistein was administered at a dose of 2 mg and 4 mg per kilogram body weight orally from day one of pregnancy till gestational day (GD) allowed for each experimental group, namely GD-13, GD-16, and GD-19.

***Tissue, blood and amniotic fluid sample collection***

Pregnant rats were weighed before being sacrificed by CO_2 _inhalation followed by cervical dislocation. Blood samples were quickly withdrawn via cardiac puncture into heparinized and sterile sample bottles. Blood samples were centrifuged at 7,000 rpm for 15 min to collect the plasma and the serum. The abdomen was wiped clean with 70% alcohol and then carefully dissected. The amniotic fluid was carefully collected into a sterile sample bottle from the amniotic fluid sack with the aid of a 15-gauge needle and syringe. The amniotic fluid was centrifuged at 3000 rpm for 15 min. The amniotic fluid supernatant was pipetted into another well-labeled sterile tube and stored at -20 ^°^C for analysis. The entire uterine implant was dissected and the placenta and fetuses separated and weighed. Placenta tissues were homogenized in Hanks buffer salt solution, centrifuged at 8,000 rpm, and the supernatant separated and stored at -20 ^°^C for analysis. Some placenta and fetuses were also stored separately in Bouin’s solution for histological analysis. All samples were collected and stored at - 20 ^°^C.

***Tissue processing for histological studies***

The placenta tissue harvested was processed using routine tissue processing procedure with graded concentration of alcohol and paraffin wax while tissues slices of 10 µm were prepared with a microtome on glass slides and stained with hematoxylin and eosin. TGCs population in the placenta tissue slides were counted under a light microscope at x40 objectives while an eyepiece with a graticule or a microscope micrometer calibration rule was used to measure the TGCs zone perimeter and layer depth.

***Transforming growth factor*** **-β1** ***assay***

Rat TGf-β1 was assayed using a TGf-β1 Enzyme-linked Immunosorbent assay kit from Elabscience according to the manufacturer’s instructions. Briefly, this assay employs a quantitative sandwich enzyme immunoassay technique that measures TGf-β1. A polyclonal antibody specific for rat TGf-β1 was pre-coated into a microplate. TGf-β1 in standards and samples was sandwiched by the immobilized antibody and biotinylated polyclonal antibody specific for TGf-β1, which is recognized by a streptavidin-peroxidase conjugate. All unbound material was then washed away and a peroxidase enzyme substrate was added according to the manufacturer’s instructions. The color development was stopped and the intensity of the color was measured with an ELISA reader.

***Alkaline phosphatase assay***

The ALP level was assayed by the autoanalyzer measurement of hydrolysis of 4-nitrophenylphosphate as described previously (25). Briefly, 0.1 ml of the enzyme preparation was diluted in 0.25 M-sucrose and incubated in 1.0 ml of a mixture containing 50 mM-glycine buffer (pH 9.8), 0.5 mM-MgCl2,6H2O, and 60 mM-substrate. 10 ml 0.02 N-NaOH was added and the extinction at 405 nm was measured against a reagent blank without enzyme.

***Statistical analysis of results***

Data were presented as mean±SEM and analyzed by parametric analysis of variance (ANOVA) followed by Tukey’s *post-hoc* test. Statistical significance was set at *P*≤0.05 while the bar chart and tables were used for data presentation.

***Ethical approval***

All the procedures adopted and used in this study were approved by the Research and Ethics Committee of College of Medicine, University of Lagos and they conformed to the Guidelines for Care and Use of Laboratory Animals in Biomedical Research (26) and were approved by the Health Research and Ethics Committee of the College of Medicine, University of Lagos (CM/HREC/11/16/071).

## Results

***Effect of in-utero exposure to genistein on the placenta and fetal weight***

**Figure 1:**
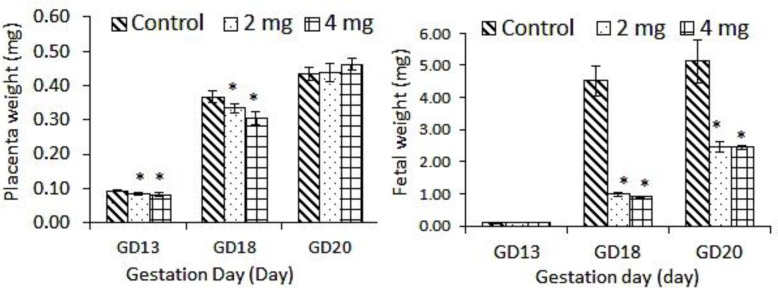
Weight of the placenta and the fetus from genistein exposed pregnancy and unexposed control at different gestation days

There was a significant decrease in the placenta weight in both 2 mg and 4 mg genistein-treated groups at GD-13 and GD-18, respectively. A significant decrease in fetal weight was recorded in both 2 mg and 4 mg genistein-treated groups at GD-20 (Figure 1).

***Effect of in utero exposure to genistein on the placenta trophoblast giant cell count, layer depth and zone perimeter in pregnancy at different gestation days***

**Figure 2:**
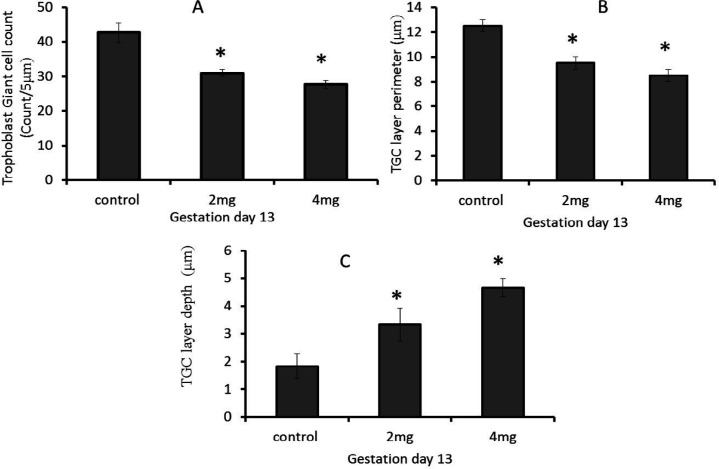
Trophoblast giant cell concentration, layer perimeter and layer depth in Genistein exposed pregnancy and control rats at gestational day 13

**Figure 3:**
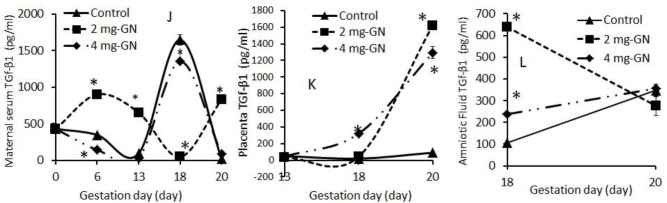
Maternal plasma, placental, and amniotic level of transforming growth factor-β1 during gestation in genistein exposed pregnancy and control

There was a significant (*P*≤0.05) decrease in TGCs count in 2 mg and 4 mg treated-groups when compared with the control groups on GD-13. There was a significant decrease in TGCs count in 2 mg genistein group and 4 mg genistein group when compared with the results of the control groups on GD-16 Day. The 4 mg treated group at GD-19 showed no significant difference whilst there was a significant decrease in TGCs count at GD-19 in 2 mg treated group. There was a significant decrease in TGCs layer perimeter in 2 mg and 4 mg treated groups when compared with the control group on GD-13. There was a significant decrease in TGCs cluster perimeter in 2 mg genistein group in comparison with control groups on GD-16, while the 4 mg treated groups showed no significant decrease in GD-16. The 2 mg genistein treated group on GD-19 showed no significant difference whilst there was a significant decrease in TGCs layer depth in the 4 mg treated group on GD-19. There was a significant increase in the depth of the giant trophoblast cell clusters in placenta tissue from pregnant rats treated with 4 mg/kg of genistein at GD-13 when compared with control (*P*≤0.05). There was a significant decrease in giant trophoblast cell cluster depth in pregnant rats of genistein treated rats in the 2 mg/kg and the 4 mg/kg groups at GD-13 when compared with control (*P*≤0.05) (Figure 2 and Figure 3, respectively).

***Effect of in utero exposure to genistein on the level of transforming growth factor-***β1*** in maternal plasma, placenta homogenate, and amniotic fluid at different gestation days***

There was a time-dependent increase (*P*≤0.05) in the placenta level of TGf-β1in all genistein exposed groups from GD-13 towards term. The maternal plasma level of TGf-β1 was significantly increased in 2 mg group between GD-0 to GD-13 while it recorded a significant reduction from GD-14 to GD-19 in all genistein groups. The amniotic fluid level of TGf-β1 was significantly increased at GD-18 in all genistein groups (Figure 3). 

***Alkaline phosphatase level in the serum, placenta and amniotic fluid from genistein exposed pregnancy and control at different gestation days***

There was a significant (*P*≤0.05) reduction in ALP level in both maternal serum and placenta homogenates in 2 mg and 4 mg genistein exposed groups at all the gestational periods (GD-13, GD-18, and GD-20) (Table 1).

*Significant compared with the control (*P*≤0.05), n=6 rats

*Significant compared with the control (*P*≤0.05)

**Table 1:** Maternal serum and placenta alkaline phosphatase level in genistein exposed pregnancy and control at different gestation days

Gestational Days	**Serum ALP (U/I)**				**Placenta ALP (U/I)**		
	**13**	**18**	**20**		**13**	**18**	**20**
Control	187.80 ± 2.80	211.80 ± 45.40	101.55 ± 1.55		1635.50 ± 128.5	1844.25 ± 323.35	2508.5 ± 58.5
							
2 mg Genistein	87.50 ± 7.50*	101.55 ± 1.55*	67.6 ± 21.70*		803 ± 47.00*	544.95 ± 4.25*	209.02 ± 0.63*
							
4 mg Genistein	124.90 ± 0.50*	118.55 ± 27.55*	40.45 ± 0.45*		301.95 ± 0.95*	1172.5 ± 94.5*	588.7 ± 11.3*

Data are represented as mean±SEM

* Significant at *P*≤0.05, n = 6

*Significant compared with the control (*P*≤0.05)

**Figure 4:**
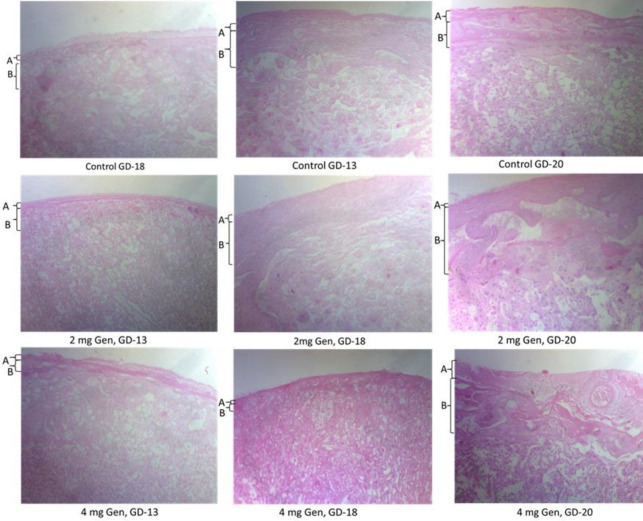
Photomicrograph of rat placenta tissue from genistein treated pregnant rat and control on gestation days 13, 18, and 20 showing the decidua layer [A], Trophoblast Giant cells layer [B]. The histological section was stained with H&E, while the picture was taken at a low magnification of x 400

## Discussion

Proper embryonic implantation is crucial for the successful establishment of pregnancy (27). TGCs are the first terminally differentiated cell type to form during embryogenesis in rodents and are of vital importance for embryo implantation, promoting an intrauterine change that allows the mother’s immune system to accept the growth and development of the embryonic implant and sustainability of the pregnancy (28). In this study, the placenta weights in the genistein exposed groups at GD-18 were significantly reduced compared with the weight recorded in the corresponding control group. The placenta is the channel through which nutrients and oxygen are supplied to the fetus, hence without this vital support, there will be a reduction in the growth of the fetus (29). The consequent significant reduction in fetal weight recorded in the genistein treated groups when compared with the control groups was therefore expected and has equally been previously reported (22, 24). Further investigation revealed a significant reduction in the TGCs population and TGCs layer thickness in the placenta morphology in genistein treated rats at GD-13 compared with the control. Other researchers have reported the apoptotic activities of genistein on the trophectoderm layer which emerges from trophoblast at the blastocyst stage of development (30). However, there was no significant change in TGCs population at GD-16 to GD-19 in genistein exposed rats indicating that this adverse effect of genistein on the TGCs proliferation occurs at the early stage of pregnancy but was reversed with the advancement in age of the pregnancy. 

Further examination revealed a significant decrease in ALP levels of serum and placenta homogenate from the genistein treated rats, suggesting that genistein interferes with ALP synthesis during pregnancy. ALP is a biomarker for the placenta function (31, 32) and its level during pregnancy is used as a measure of the health status of the placenta in carrying out its statutory role in the sustenance of pregnancy (32-34). An increase in maternal serum and placenta ALP has been linked with the burrowing activities of TGCs into the uterine wall and subsequent implantation of the blastocytes with further formation of blood vessels (23, 24, 35–38). Thus, the reduced population of the TGCs recorded in the genistein treated rats especially at the early stage of gestation may have precipitated the reduction in placenta ALP level as recorded in this experiment. We have reported a significant reduction in the inflammatory activities at a similar period in pregnancy exposed to genistein as indicated by a reduction in the plasma C-reactive protein level (22, 23), with precipitated adverse consequences on oocyte fertilization, implantation, and survival of the embryonic implants.

Aberrant effect of genistein on the functional anatomy of the placenta further revealed shrinkage in the TGCs layer and perimeter, an indication of anti-proliferation of the TGCs within the placenta. This was further buttressed with the significant reduction in the weight of the placenta in all genistein exposed rats from the early stages of the pregnancy towards GD-18. In this study, the placenta TGf-β1 level and its concentration across the maternal serum and the amniotic fluid were significantly altered. The highest level was recorded in the placenta followed by the maternal serum and the amniotic fluid. The TGf-β1 isoform is a polypeptide secreted by several cell types with such identified functions as pro-inflammatory functions (39), immune suppressor function (40), and an anti-cell proliferation function (41). There was a significant increase in the placenta TGf-β1 in all genistein exposed rats but most especially in the 4 mg group at GD-18 and in the two genistein groups towards term. The observed increased level of TGf-β1 with anti-cell proliferation functions (41) may explain the recorded decreased placenta cell growth and weight as recorded in all genistein exposed rats in this study. The recorded decrease in the maternal serum level of TGf-β1 in the 2 mg genistein group could not be fully explained but may not be unconnected with the selectivity in the activity of genistein with higher affinity for ER-β than ER-α (42) which is also dose-dependent. Thus, genistein appears to promote increased secretion of placenta TGf-β1 which might have precipitated increased apoptosis of the TGCs with consequent reduction in their population and thus a reduction in placenta weights. This corroborated previous report where disruption in the TGf-β1 signaling was found to alter trophoblast differentiation in preeclampsia (43). The increase in placenta TGf-β1 recorded in this study may thus not be unconnected with the apoptosis and shrinkage in the population and size of TGCs in placenta tissue samples from all genistein exposed rats. 

## Conclusion

Genistein appears to cause degeneration of placenta tissue via increased levels of TGf-β1 and ALP with consequent apoptosis of TGCs leading to a decrease in its population and a reduction in placenta weight.
